# Ten tips to preserve and reuse water in nephrology

**DOI:** 10.1093/ckj/sfag208

**Published:** 2026-06-18

**Authors:** Susi Knöller, Catherine Weber, Mohamed Ben Hmida, Rui Lucena, Faissal Tarrass, Stephan Segerer

**Affiliations:** Gesundheit Nord, Klinikum Bremen-Mitte, Department of Nephrology, Bremen, Germany; McGill University, Department of Nephrology, Montreal, QC, Canada; Hedi Chaker Hospital, Department of Nephrology, Sfax, Tunisia; Research Laboratory of Renal Pathology LR19ES11, Faculty of Medicine, University of Sfax, Sfax, Tunisia; International Organization for Standardization (ISO), Geneva, Switzerland; Fesenius Medical Care Deutschland, Department Value Stream WTS, Bad Homburg, Germany; Center of Hemodialysis 2 Mars, Casablanca, Marocco; Kantonspital Aarau AG, Department of Nephrology, Aarau, Switzerland

**Keywords:** circular water management, green dialysis, sustainable nephrology, water conservation, water reuse

## Abstract

**Background:**

Water scarcity is an escalating global challenge with growing relevance for healthcare systems. Nephrology is particularly affected, as dialysis is a life-sustaining yet highly water-intensive therapy. Climate change, droughts, and infrastructure vulnerabilities increasingly threaten reliable water access, exposing the fragility of water-dependent kidney replacement therapies.

**Methods:**

We reviewed the available evidence and practical experience on water use in nephrology and dialysis care, with a focus on feasible strategies to reduce water consumption without compromising patient safety or quality of care.

**Results:**

We identify key domains of water use in dialysis and nephrology practice and present 10 practical, evidence-informed tips to reduce water consumption. These measures span system-level approaches, technological considerations, staff and patient engagement, behavioral change and are applicable across diverse resource settings.

**Conclusions:**

Integrating water stewardship into routine nephrology practice is essential to enhance the long-term resilience of dialysis services. Proactive water conservation represents a clinically relevant, ethical, and achievable component of sustainable kidney care.

## Graphical Abstract

**Graphical Abstract:**
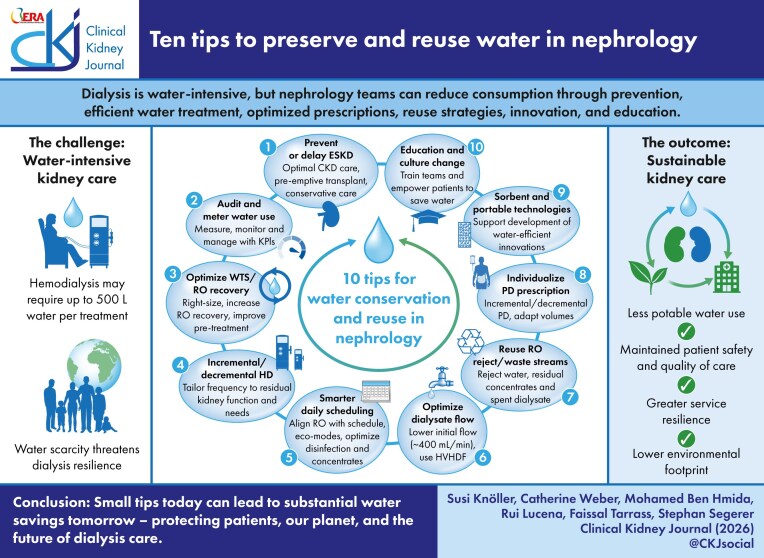

## INTRODUCTION

“Water is the root of everything” (Thales of Miletus; circa 624–547 BC). It is essential for biological processes at the cellular and systemic level, underpins ecosystem stability, and is a cornerstone of public health and global development [1]. Yet access to clean and sufficient water is increasingly jeopardized. Global data reveal significant geographic disparities in freshwater withdrawals, with per-capita usage exceeding 1000 m³ annually in certain high-income regions, while remaining substantially lower in many low- and middle-income countries (Table 1) [2]. The United Nations estimates that nearly two billion individuals lack access to safely managed drinking water, and billions more experience seasonal water scarcity, with drought-related crises emerging as a recurrent global threat [2]. In this context, the water footprint of healthcare warrants urgent attention.

**Table 1:** Water withdrawal in different countries per capita.

Region	Approx. freshwater withdrawal per capita ( ${{\mathrm{m}}}^3/$person/year )
Sub-Saharan Africa	∼300–600 (lower than global average)
Europe	∼600–900 (moderate to high)
Middle East & North Africa	$\sim $ 500–800 (variable, often mid-range)
North America	>1000 (among the highest)

Compiled from AQUASTAT and the World Bank.

In nephrology, life-sustaining hemodialysis ranks among the most water-intensive medical interventions [3]. At a dialysis fluid flow rate of 500 ml/min, ∼120 l of dialysis water are delivered per 4-h treatment; however, due to reverse osmosis (RO) recovery limitations and additional operational requirements (e.g. priming, rinsing, and disinfection), the total facility water draw per treatment can approach 500 l. For a patient treated three times weekly, this equates to ∼78 m³ per patient-year, corresponding to roughly 8%–26% of annual per-capita freshwater withdrawals reported across regions (Table 1), depending on local context. Globally, hemodialysis alone is estimated to consume ∼265 million m³ of water annually [4].

The number of patients receiving kidney replacement therapy (KRT) is projected to exceed five million by 2030; therefore, the environmental footprint of dialysis must be seriously addressed by the nephrological community [5].

Climate change, drought, and infrastructure vulnerabilities increasingly impact health systems worldwide. Dialysis services have already been disrupted in settings facing acute water shortages and natural disasters, underscoring the fragility of water-dependent therapies [6].

Water availability, infrastructure, and regulatory frameworks for water reuse vary substantially across regions; consequently, the feasibility and prioritization of specific interventions (e.g. reuse of RO reject water, gray-water applications, potable vs. non-potable reuse) will differ by local context.

This article presents 10 practical, evidence-informed tips to reduce water consumption in nephrology practice and beyond, supporting resource stewardship and the long-term resilience of KRT under increasing environmental constraints.

### Tip 1: Reduction of water by avoiding dialysis

Preventing the progression of chronic kidney disease (CKD) to end-stage kidney disease (ESKD) can avoid or delay water use for dialysis. In proteinuric CKD, strong evidence supports a multi-targeted approach including renin–angiotensin–aldosterone system inhibition, SGLT2 inhibitors, mineralocorticoid receptor antagonists, and more recently GLP-1 receptor agonists to slow disease progression [7].

The most effective water-saving intervention in those who develop ESKD is pre-emptive transplantation. Early identification of eligible CKD patients enables timely education, living donor recruitment, and completion of transplant evaluation. Despite clear benefits—including improved survival, enhanced quality of life, and avoidance of dialysis-related complications, registry data show pre-emptive transplantation accounts for only 4% of European transplants and 3% of transplants in the United States [8]. A 10% increase in kidney transplantation activity in Europe (corresponding to ∼2500 additional transplants annually, based on ERA Registry data [9]) could translate into substantial water savings, roughly 195 million liters of water annually. This simplified estimate assumes that these additional transplant recipients would otherwise have required maintenance dialysis for a full year.

Many European countries have legislated opt-out or presumed consent policies for organ donation, with the notable exception of Germany. However, transplantation access still varies substantially across Europe, indicating that policy frameworks alone are insufficient and that broader systemic, organizational, and cultural factors also shape transplantation rates [10, 11].

In ESKD patients where transplant is not an imminent option and/or the patient is ineligible, initiation of KRT should be guided by symptoms and biochemical parameters rather than an arbitrary eGFR threshold [12, 13]. This water-saving approach is supported by a large, randomized trial showing no increase in mortality with delayed initiation [14].

While not a water-saving strategy per se, it is important to evaluate the appropriateness of initiating KRT in certain patients in the context of their personal preferences [15]. Many countries endorse conservative care for older adults and those with significant comorbidities [16] however widespread adoption of this patient-centered approach rather than de-facto KRT initiation will require substantial re-alignment of incentives.

All recommendations in this context must be guided primarily by patient-centered outcomes, clinical appropriateness, and ethical considerations; potential resource savings should be regarded as a secondary benefit rather than a driver of decision-making.

### Tip 2: Assess the current state of water utilization in your practice

**Figure 1:**
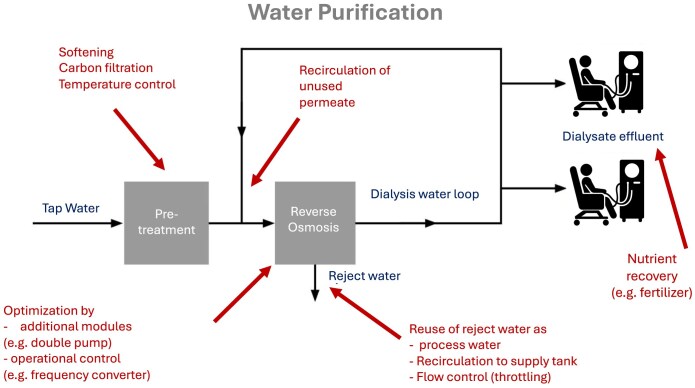
Schematic overview of a contemporary dialysis WTS and potential intervention points for water conservation. The WTS comprises three main stages: pre-treatment of municipal feed water, RO as the primary purification step, and distribution of purified water via the loop to dialysis machines. Potential intervention points to improve water efficiency and enable water reuse, where permitted, are highlighted throughout the diagram. The schematic is simplified and does not include all monitoring and safety components.

**Figure 2:**
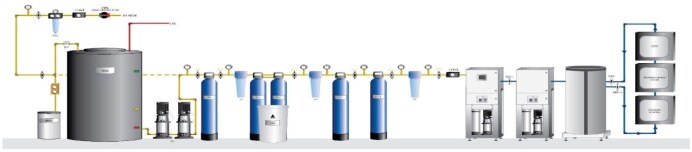
Technical components of a contemporary dialysis WTS.The WTS comprises three functional phases: (1) inlet and pre-treatment of municipal feed water, including protection devices and processes such as filtration, softening, and carbon treatment; (2) primary treatment based on RO, with optional additional treatment depending on local conditions; and (3) distribution of treated water to dialysis machines and related systems via one or more distribution loops. Monitoring, control, and alarm functions are integrated throughout the system. Reject water and wastewater may be discharged or collected for non-clinical reuse, subject to local regulations and water quality requirements.

The annual per-capita freshwater withdrawals exhibit significant regional variation (Table 1). An initial step toward reduction involves comprehending and documenting the primary sources of water consumption through a professional water audit. In nephrology practice, immediate actions such as repairing leaks can decrease water usage by 10%–15%, while modernizing the water treatment system (WTS, Figs 1 and 2), including implementing reuse strategies, may achieve reductions of 30%–60% [17]. Audits should be grounded in direct measurements. For this purpose, water meters are necessary at critical points, including the unit inlet, WTS inlet and outlet, and RO inlet [18]. This facilitates differentiation between fixed and treatment-related consumption, early detection of losses, identification of saving opportunities, and evaluation of interventions. To avoid a “one-size-fits-all” approach, audits should be interpreted within the local context. Water availability, tariffs, infrastructure resilience, and regulatory frameworks (including permitted reuse routes and water quality requirements) differ substantially across countries and regions. Consequently, uniform key performance indicators (KPIs) should be used primarily for internal trending, while cross-country benchmarking should be interpreted cautiously and adjusted for local determinants (e.g. RO recovery, disinfection practices, and whether water reuse is permitted). Wastewater or reject-water reuse policies and purity requirements vary and may determine which options are feasible (e.g. non-clinical reuse vs. restrictions on reuse in healthcare settings). Audits should therefore pair quantitative KPIs (e.g. liters per treatment; RO recovery; reject ratio) with a brief site “context profile” (water-scarce vs. water-rich setting; regulatory constraints; reuse permissions) to ensure meaningful comparisons and realistic targets. Finally, professional audits can also raise awareness beyond the clinic, supporting staff education, behavior change, and a culture of continuous improvement.

### Tip 3: Optimizing the water treatment system (WTS) to reduce dialysis water consumption

Optimizing the WTS (Fig. 1) is among the most effective strategies to reduce dialysis water consumption and should be addressed at the design stage. Fixed sizing rules, typically set at 50–60 l per station per hour, can result in oversized systems, whereas actual demand may be lower, ∼30 l per station per hour, particularly in centers delivering post-dilution high-volume hemodiafiltration (HVHDF) with accurately prescribed flows. As new units often operate at partial capacity during initial stages, demand-based sizing and modular or scalable concepts can more accurately align with actual requirements, thereby reducing capital expenditures and minimizing water and energy consumption.

RO serves as the primary purification step, with the central metric of water efficiency being RO recovery (yield), defined as the proportion of RO feed water converted to permeate [19]. Theoretical feed-water draw is proportional to recovery (feed ≈ permeate/recovery): at ∼50% recovery, around 240 l of tap water are needed to produce 120 l of dialysis water. In contrast, modern systems, where conditions allow recoveries up to ∼85%, would require about 141 l for the same permeate volume [19]. Reported per-treatment facility water use can be substantially higher because it reflects a broader system boundary and includes RO reject and fixed operational losses (e.g. priming, rinse/disinfection, inter-sessional idle phases, WTS pre-treatment backwash/regeneration and rinse/disinfection). Such higher figures often reflect lower-efficiency RO configurations (e.g. recovery <70%) in addition to these fixed losses. With higher-efficiency RO operation and optimized design/operation, where validated and permitted by feed-water quality and local requirements, total facility water draw can be significantly reduced, with some centers reporting <250 l per treatment [18].

Increasing recovery raises concentration factors and can promote scaling and biofouling; therefore, recovery should be optimized within a validated operating envelope. Regular specialist evaluation is recommended, including trend analysis of rejection/normalized performance, fouling/scaling risk assessment, and verification of disinfection and flushing cycles [18]. Achievable recovery and stability depend on feed-water temperature/seasonality, unit size/utilization, and tap-water quality/pre-treatment configuration [18].

Enhancing inlet-water quality is a crucial preventive measure. Dialysis pre-treatment is typically configured based on the local tap-water chemical and microbiological profile and the RO inlet-water specifications, and commonly includes multimedia filtration, activated carbon and softening (with fine filtration as appropriate). Microfiltration/ultrafiltration may be considered in selected settings to reduce RO fouling and cleaning frequency [18, 20].

Further savings can be achieved by optimizing pretreatment operating cycles: backwash/regeneration of multimedia filters, softeners, and carbon filters often follow fixed schedules and can consume more than 1000 l per cycle; condition-based triggers can reduce unnecessary water use [19].

Upgrading outdated, low-recovery RO systems to modern high-efficiency units can yield substantial savings (Tables 2 and 3); Bendine *et al*. reported reductions of nearly 50% in routine practice [21].

**Table 2:** Ten tips to preserve and reuse water in Nephrology practice.

Tip	Take-home message	Impact (water savings)	Patient benefit
Tip 1	Prevent or delay ESKD progression via optimal CKD management, pre-emptive transplantation, and considering conservative care in accordance with patient preferences.	Very strong (∼6000 l per patient-month)	Strong (avoids dialysis, improves survival/QoL)
Tip 2	Conduct a professional water audit and install meters to measure consumption and create KPIs for management.	Moderate to low (reductions of 10%–15% possible)	Neutral (safety maintained)
Tip 3	Optimize water treatment system sizing, RO recovery, pre-treatment, and monitoring to maximize efficiency.	Strong (savings >35 lper treatment; up to 50% with upgrades)	Neutral (safety maintained)
Tip 4	Use incremental or decremental HD tailored to residual kidney function to reduce treatment frequency.	Strong (∼2000 l per patient-month)	Strong (preserves QoL, avoids overtreatment)
Tip 5	Align RO operations with schedules, use machine eco-modes, optimize disinfection, and centralize concentrates.	Moderate (reduces fixed/operational waste)	Neutral (safety maintained)
Tip 6	Optimize initial dialysate flow rate (target ∼400 ml/min, Qd/Qb ≤ 1.3) and consider high-volume HDF for efficiency.	Moderate to strong (savings ∼25 l per treatment)	Neutral to positive (equivalent clearance in HDF)
Tip 7	Reuse RO reject water (e.g. irrigation, toilet flushing), harvest residual concentrates for non-sterile uses and recycle spent dialysate.	Strong (30%–50% reduction in feed water use)	Neutral (non-clinical use)
Tip 8	Tailor PD prescription (incremental/decremental) to patient needs and optimize bag volumes to reduce direct use.	Moderate to strong (varies with prescription)	Strong (personalized therapy)
Tip 9	Support development of sorbent-based dialysis systems that regenerate dialysate (e.g. NeoKidney, AWAK PD).	Potentially very strong (uses only 2–7 lper session in prototype systems)	Strong (portability, home use)
Tip 10	Implement structured education on water stewardship for all levels to drive cultural and behavioral change.	Potentially strong (foundational for all tips)	Indirect (sustainable care model)

Abbreviations: CKD, chronic kidney disease; ESKD, end-stage kidney disease; HD, hemodialysis; HDF, hemodiafiltration; KPI, key performance indicator; PD, peritoneal dialysis; Qd/Qb, dialysate-to-blood flow ratio; QoL, quality of life; RO, reverse osmosis; WTS, water treatment system.

**Table 3:** Impact–effort matrix of 10 water-saving tips in nephrology and dialysis practice.

Impact ↓/Effort →	Low effort	Medium effort	High effort
Low to moderate water-saving impact	Tip 2 water audit & measurements Tip 5 optimizing daily program Tip 6 dialysate flow optimization		
High water-saving impact		Tip 3a operational optimization (RO recovery, pre-treatment cycles, monitoring-based adjustments Tip 4 incremental/decremental HD Tip 7a RO reject water reuse Tip 8 incremental/ decremental PD	Tip 1 preventing ESKD avoiding dialysis Tip 3b refurbishment/upgrades of WTS Tip 7b concentrates and spent dialysate reuse Tip 9 reduction of water via new technology Tip 10 Education

Tips are grouped qualitatively according to their implementation effort and their potential water-saving impact. The matrix provides a conceptual overview to support prioritization without implying quantitative ranking. WTS, water treatment system.

RO reject water may also be reused for non-clinical purposes where regulations and water quality permit [22, 23].

Continuous monitoring of inlet-water quality and RO parameters remains essential; during periods of deteriorating municipal water quality, temporarily conservative settings may be required, but surveillance enables timely return to high-efficiency operation. Finally, liters per treatment or percentage savings should be interpreted cautiously because they combine fixed and treatment-related consumption; high-throughput units may appear more efficient partly due to dilution of fixed consumption rather than true performance differences.

### Tip 4: Conserving water by using incremental and decremental hemodialysis

For patients with substantial residual kidney function (RKF), incremental hemodialysis (<3 sessions/week) is increasingly adopted, supported by studies demonstrating feasibility and clinical safety [24, 25].

In addition to RKF (urine output ≥500 ml/day or residual urea clearance ≥2–3 ml/min), eligible patients should be clinically stable and free from significant uremic symptoms, hypervolemia or hyperkalemia, and compliant with frequent monitoring to establish continuing candidacy for incremental therapy. Further practical considerations include low interdialytic weight gain and limited ultrafiltration requirements, which may indicate sufficient endogenous fluid and solute control and support the safe implementation of incremental strategies [25].

A recent multicenter randomized trial from Spain (*n* = 150 patients) reported 44% fewer treatment sessions in the incremental arm compared to conventional thrice-weekly dialysis, with no significant difference in clinical outcomes like GFR decline, hospitalization, mortality, or quality of life at 12 months [26].

Several large, randomized trials (TwoPlus Trial NCT05828823, IHDIP Trial NCT03239808) are underway and could drive a paradigm shift to avoid unnecessary water-intensive dialysis sessions in selected patients thereby not only reducing resource utilization but also potentially sparing patients time-consuming treatments and affording them more time in daily life.

Nutritional strategies aimed at preserving RKF and prolonging time on incremental therapies should be considered in motivated patients. A small RCT in incident ESKD patients (mean eGFR 6.5 ml/min/1.73 m²) showed that a low-protein diet (0.6 g/kg/day + keto acid supplementation) combined with once-weekly HD preserved RKF and urine volume better than twice-weekly HD with a normal protein diet at 6 and 12 months, without affecting survival [27].

On the other end of the spectrum, a decremental approach in frail ESKD patients near the end of life may provide sufficient clearance and volume removal even without significant RKF with the goal of maximizing quality of life, rather than an “all or none approach.” Evidence evaluating decremental dialysis is extremely limited.

### Tip 5: Reduce water usage by optimizing the daily program in the dialysis ward

The daily operations within the dialysis ward significantly impact water consumption, and minor adjustments can effectively reduce unnecessary waste. It is advisable to synchronize the start and stop times of RO systems with the treatment schedule, as premature activation or extended operation post-treatment can lead to increased permeate production, flushing, and fixed water usage. By coordinating these routines, baseline water consumption can be minimized while ensuring hygienic safety within validated operational parameters [18].

The primary consideration is microbiological safety; thus, water-saving strategies must not elevate the risk of stagnation or contamination. Where feasible, dialysis water circuits and distribution loops should remain permanently connected and managed as closed systems to minimize disconnections and dead space, while maintaining appropriate recirculation during idle periods. Dialysis machines should be powered on shortly before session commencement, utilizing standby or “eco-flow” modes until patient connection to reduce dialysate flow and circulation while maintaining readiness [28].

Proactive microbial control is essential to limit microbial growth, biofilm formation, and biofouling. Although some biofilm formation is inevitable, excessive accumulation that hinders routine achievement of action levels for microorganisms and endotoxins compromises system operation [29]. It is crucial to minimize biofilm development from the outset, as established biofilm can be challenging, if not impossible, to eradicate.

Disinfection protocols should be based on a validated, risk-based approach rather than rigid schedules [19]. During validation (operational/performance qualification), strategies should be optimized based on utilization patterns, microbiological trends, and strict adherence to manufacturers’ instructions for use. Where applicable, scheduled thermal (hot) disinfection of machines and/or the distribution loop should be integrated; the required frequency is variable and should be defined during validation and adjusted through trend analysis and system events. Given that biofilm growth is contingent on nutrient availability, control should not rely solely on biocides, as some oxidizing agents may increase bioavailable organics. For machines temporarily out of service, it is important to follow manufacturer guidance while avoiding unnecessary cycles that increase water and energy consumption [28].

Recent studies indicate that in-house preparation of concentrates from powder with central distribution may reduce overall resource use compared with canisters or bags, which largely transport water. By avoiding pre-filled liquid concentrates and using on-site dialysis water instead, material demand (especially plastics and transport-related inputs) is reduced, which likely also translates into lower upstream water consumption associated with manufacturing and logistics. While direct quantification of water savings remains limited, this approach suggests a more water-efficient supply chain overall [30–32].

### Tip 6: Conserve water through the optimization of dialysate flow

Dialysate flow rate (DFR) is often overlooked in hemodialysis prescriptions, yet significantly impacts water utilization. It primarily influences the clearance of small, water-soluble solutes, while removal of protein-bound toxins is largely independent of DFR and depends on other factors such as membrane characteristics and treatment modality. In clinical practice, DFR should be interpreted in relation to blood flow rate (Qb), as increasing DFR beyond a certain ratio provides diminishing returns in solute clearance [33].

In a small cross-over study conducted in Colombia, 46 prevalent hemodialysis patients weighing <65 kg were randomized to a DFR of 400 ml/min versus 500 ml/min. [34]. After 4 weeks, there was no difference in clearance (Kt/V 1.57 vs. 1.59, *P* = .45). A follow-up study from this group using registry data in 71 patients (32% DFR 400 ml/min and 68% DFR 500 ml/min) reported no difference in mortality at 2 years, even after adjustment for multiple clinical variables [35].

An observational study from Marocco in 33 prevalent HD patients with weight <65 kg compared autoflow of 1.3 (automatically adjusts dialysate flow to 1.3× blood flow, equivalent to DFR ∼400 ml/min) to DFR 500 ml/min and 700 ml/min. The achieved Kt/V with online clearance monitoring was 1.49, 1.50, and 1.52, respectively, with a statistical difference observed only between 500 and 700 ml/min [36].

HVHDF offers a mortality benefit over high-flux HD when blood flow rates >350 ml/min and substitution volumes above 23 l are achieved [37]. In hemodiafiltration, part of the convective volume is delivered as substitution fluid, which partly replaces dialysate and may therefore influence overall dialysate requirements [37]. HVHDF may also reduce water use: a simulation study showed similar clearance (spKt/V = 1.65) with HVHDF versus HD using less dialysate (99 l vs. 125 l); the authors confirmed their model by applying the parameters to real-world retrospective data (DFR ∼430 ml/min and the auto-substitution function which avoids excessive hemoconcentration within the dialyzer by continuously adapting substitution flow) [33]. Although these observations are promising, they require further validation.

Although the evidence base is limited, it appears reasonable to preset DFRs at a lower level (e.g. ∼400 ml/min in patients with high-performing vascular access) and increase flow according to clearance targets and patient well-being under close monitoring. Individualization remains essential, prioritizing patient benefit through consideration of patient-specific factors (i.e. body volume) and HD parameters (blood flow rate, treatment time, HVHDF and automated functions).

### Tip 7: Reuse water, electrolyte solutions, and dialysate

Sustainable water management in hemodialysis requires a multi-stream approach, with RO reject water offering a major conservation opportunity. Accounting for 30%–40% of feed water, it is characterized by elevated salinity while remaining low in microbiological contamination [38]. Its reuse depends mainly on electrical conductivity (EC): water with EC <1500 µS/cm is suitable for irrigation, while EC 1500–2400 µS/cm can be used for non-potable purposes such as toilet flushing and surface cleaning [39]. Although installing systems for collection, storage, and redistribution requires initial investment, the continuous reduction in potable water use supports long-term economic viability [40]. Another often overlooked waste stream is residual electrolyte solution from concentrate-canisters. Sodium bicarbonate cartridges and acid concentrate containers retain substantial volumes of saline solution, with 1.5–2.5 l commonly discarded from each 4.5 l acid container, representing both financial loss and environmental burden [41]. While non-sterile, these fluids have controlled composition and are suitable for non-sterile reuse, such as initial machine rinsing or sanitary flushing [41, 42].

This approach diverts liquid from medical waste, conserves potable water and supports sustainability and has been proposed for non-sterile reuse applications, although implementation is often limited by regulatory and safety considerations [41, 42].

The most complex but promising stream is spent dialysate. Beyond material and water reuse, thermal energy recovery from spent dialysate represents an emerging strategy to further reduce the environmental footprint of dialysis facilities [40]. Including integration of heat exchangers and heat pumps may allow recovery of waste heat from dialysate effluent, although implementation depends on local infrastructure and economic feasibility [40]. Although direct reuse is contraindicated due to its uremic toxin content, advanced treatment using RO or nanofiltration can remove over 95% of contaminants, including salinity and emerging pollutants, producing high-quality water for non-potable reuse [43].

In addition, its nutrient content, rich in ammonia nitrogen and phosphates, enables recovery of struvite fertilizer through crystallization [44]. This integrated management of spent dialysate can reduce the environmental impact of dialysis by 30%–50%, transforming the largest waste stream into a source of water and nutrients [43].

### Tip 8: Reduction of water use in peritoneal dialysis (PD)

Water consumption in peritoneal dialysis (PD) can be divided into direct use (sterile dialysate volume) and indirect use related to plastic production and dialysate manufacturing [45].

Direct water use depends on the prescription: CAPD with 4 × 2 l exchanges requires ∼2920 l per year, while APD with 2 × 5 l nocturnal exchanges plus 2 l daytime dwell uses about 4380 l annually.

Indirect water consumption is substantial. Approximately 180 l of water are required to produce 1 kg of plastic [4]. A 2 l PD bag contains around 0.155 kg of plastic, corresponding to ∼28 l of water per empty bag. Including bag and content, annual water use amounts to roughly 44,000 l for CAPD and 63,000 l for APD. Water use for dialysate production itself is provider-dependent and currently undisclosed, likely because production steps are distributed across suppliers [4]. Data from major Swiss PD fluid manufacturers could not be published, but plastic production was identified as the dominant contributor (personal communication). Consequently, the overall difference in water use between PD and HD may be smaller than assumed but remains uncertain [4]. A recent study using life cycle assessment described significantly higher estimates than the above mentioned [45].

Direct water use can be reduced by individualizing PD prescriptions, adjusting bag number and volume to RKF, fill volume, and APD settings. Incremental PD may further lower environmental impact [45].

For enhancing resilience, local manufacture of PD solutions and production could eliminate packaging and transport and would likely offer the greatest water savings; unfortunately, it is currently not commercially available [46].

### Tip 9: Reduction of water via new technology

Sorbent-based dialysis systems regenerate dialysate using sorbent cartridges. Dialysate is continuously recirculated through a cartridge containing urease and ion-exchange media that remove uremic toxins, enabling dialysis in a closed-loop circuit using only a few liters of dialysate [46, 47].

Several sorbent-based devices are currently under development, including the NeoKidney (Neokidney Portable Hemodialysis System) and the WAK (Wearable Artificial Kidney) for HD, as well as the AWAK PD (Automated Wearable Artificial Kidney for Peritoneal Dialysis) and WEAKID (Wearable Artificial Kidney for Peritoneal Dialysis) for PD. While these prototypes suggest a potential for substantial reductions in water consumption, they are not yet implemented in clinical practice, and robust data on their real-world impact remain limited [48–51]. The overall sustainability of sorbent-based systems cannot yet be fully assessed, as the manufacturing and supply-chain water footprint of sorbent cartridges remains insufficiently characterized. A 2016 proof-of-concept study reported that the WAK could deliver prolonged dialysis operation using ∼375 ml of dialysate in a recirculating system [48].

The lightweight portable NeoKidney uses ∼4.5 l of dialysate per treatment session through sorbent-based regeneration. Reported data suggest solute clearance comparable to that achieved with conventional thrice-weekly hemodialysis. A first-in-human study indicated promising efficacy [49].

The AWAK PD continuously recirculates regenerated dialysate at ∼2 l per hour, enabling prolonged PD with reduced dialysate requirements. Initial clinical studies suggested acceptable safety and feasibility, and the device has received US Food and Drug Administration Breakthrough Device designation [50].

The WEAKID recirculates ∼7 l of dialysate overnight and has been reported to achieve improved toxin clearance compared with conventional PD. A first-in-human trial is currently ongoing [51].

Although the available data are promising, important technical and implementation challenges remain, several studies are now dated, and none of these systems has yet reached routine clinical practice.

### Tip 10: Education to reduce water consumption

The transition to environmentally responsible kidney care requires structured education at all levels. Recent initiatives in green nephrology emphasize multidisciplinary approaches, including dedicated curricula and training modules to increase awareness of environmental impacts and support sustainable clinical decision-making [52, 53]. Team-based interventions involving physicians, nurses and technical staff can facilitate the implementation of resource-saving practices in dialysis units [52, 54]. In addition, structured patient education—particularly regarding home dialysis—has been associated with increased uptake of home-based therapies and may contribute to a lower environmental footprint [55]. Fostering awareness, shared responsibility and behavior change is essential to translating these principles into routine practice. Engaging both healthcare professionals and patients supports the adoption of resource-efficient strategies and aligns clinical decision-making with long-term sustainability goals. Education therefore represents a key component of sustainable nephrology and the long-term resilience of dialysis care [55].

## OUTLOOK

**Figure 3:**
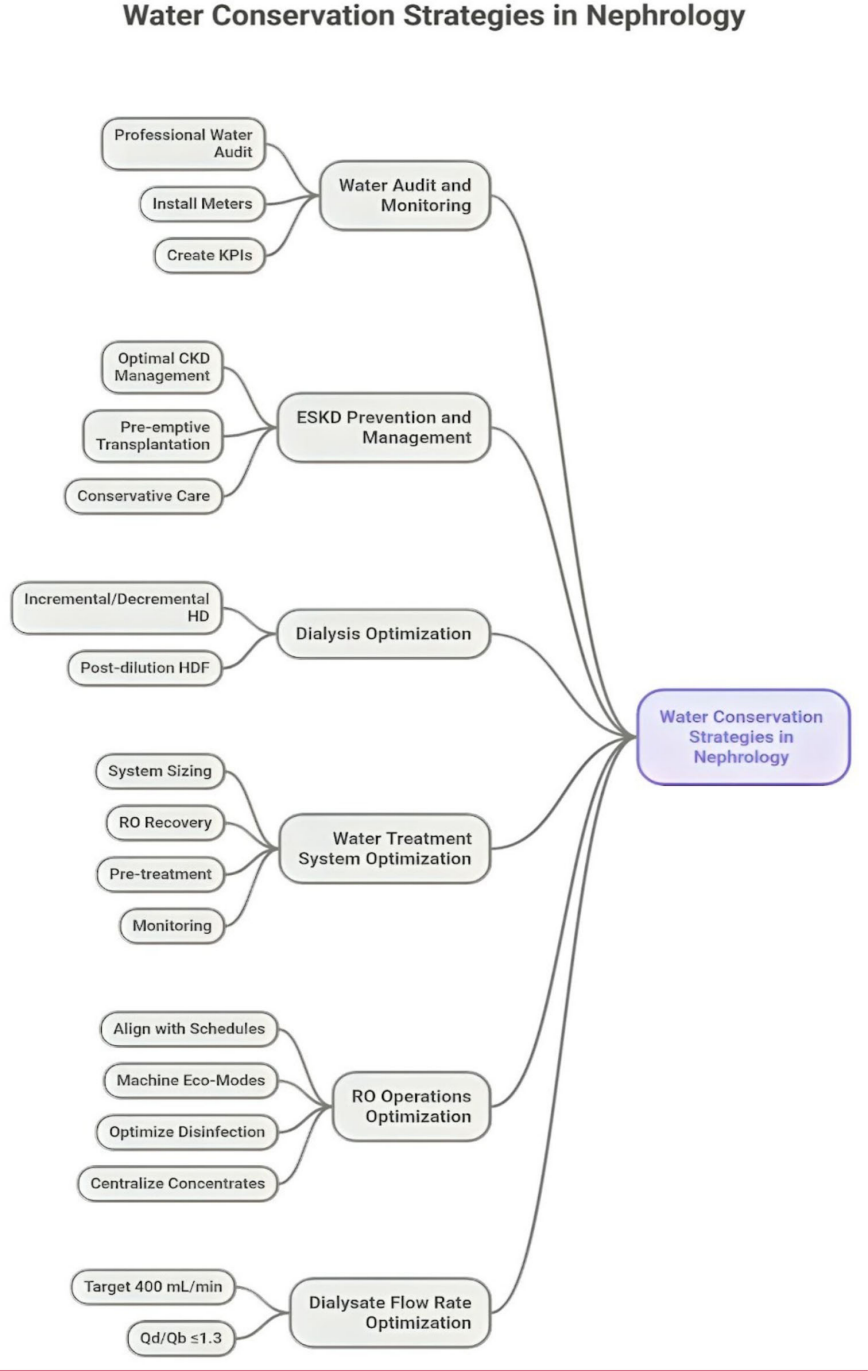
Summary of the 10 tips for water conservation and reuse.

**Figure 4:**
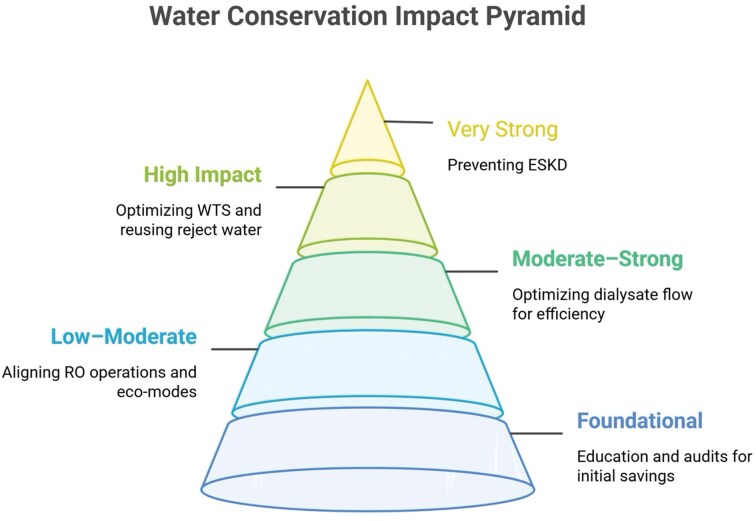
Illustration of the relative potential impact of different water-saving strategies in nephrology, ranging from foundational operational measures to high-impact interventions such as preventing ESKD and avoiding dialysis.

This paper aimed to summarize practical approaches to water preservation, encourage greater efforts in water saving and expand awareness of available strategies. Fig. 3 provides an overview of the proposed tips, while Tables 2 and 3 and Fig. 4 summarize the water-saving potential and semi-quantify the effort required to implement these measures in routine practice.

We suggest starting with small, rapidly achievable goals. Even modest reductions in water use can accumulate substantially over time, given the high number of hemodialysis treatments performed. We thank readers for their commitment to more sustainable nephrological care and hope this manuscript supports the reduction of water consumption in daily clinical practice.

## Data Availability

No new data were generated or analyzed in support of this research.
